# Surgical resection of advanced non-small cell lung cancer after a response to EGFR-TKI: presentation of two cases and a literature review

**DOI:** 10.1186/s13019-017-0668-3

**Published:** 2017-11-23

**Authors:** Yoko Yamamoto, Ken Kodama, Tomohiro Maniwa, Masashi Takeda

**Affiliations:** 1 Department of Thoracic Surgery, Yao Municipal Hospital, 1-3-1 Ryuge-cho, Yao City, Osaka 581-0069 Japan; 2 Department of Pathology, Yao Municipal Hospital, Yao City, Osaka Japan

**Keywords:** Non-small cell lung cancer, EGFR mutation, EGFR-TKI, Surgery

## Abstract

**Background:**

The usefulness of residual tumor resection after epidermal growth factor receptor tyrosine kinase inhibitor (EGFR-TKI) treatment remains unclear. We describe two patients who underwent residual tumor resection after responding to EGFR-TKIs for advanced non-small cell lung cancer (NSCLC) harboring EGFR gene mutations, along with a review of the literature.

**Case presentation:**

The patient in Case 1 was a 72-year-old female non-smoker who was initially diagnosed with T2aN2M0, stage IIIA adenocarcinoma harboring an EGFR exon 21 L858R mutation. After 8 months of gefitinib therapy, a marked radiologic response was noted, and right upper lobectomy with systemic lymph node dissection was performed. The patient developed brain metastasis despite continuous gefitinib therapy. The patient in Case 2 was a 68-year-old female non-smoker who was initially diagnosed with T3N2M0, stage IIIA adenocarcinoma and an extensive pulmonary thromboembolism. After 3 months of therapy with afatinib and anticoagulants, a marked radiologic response and symptom relief were achieved. We then performed right bilobectomy with systemic lymph node dissection. She developed bone metastasis despite postoperative afatinib therapy.

**Conclusion:**

The timing and validity of salvage surgery for residual lesions remain unclear when TKIs are offered as first-line therapy to patients with advanced NSCLC. In our two cases, surgery was performed without any complications. Surgical resection of the residual tumor might contribute to good local control. The accumulation of more clinical data is needed to further investigate the role of surgery in patients with advanced NSCLC harboring EGFR gene mutations.

## Background

Lung cancer remains the leading cause of cancer-related mortality throughout the world [1]. Radical surgery is not generally indicated for advanced lung cancer, and systemic chemotherapy is the mainstay of treatment. In the past decade, several molecular-targeted agents, such as epidermal growth factor receptor tyrosine kinase inhibitors (EGFR-TKIs), have emerged and are currently used in the treatment of advanced non-small cell lung cancer (NSCLC). EGFR-TKIs have been shown to play a significant role in treatment, and are considered to be a first-line treatment of choice for advanced EGFR mutation-positive NSCLC. EGFR-TKIs, which have reduced toxicity, are able to induce a stronger and a more rapid response; however, most patients eventually develop tumor progression due to acquired resistance, and the progression-free survival period after the initiation of first-line EGFR-TKI treatment has been reported to be less than 1 year [2]. The role of residual tumor resection after EGFR-TKI treatment for advanced NSCLC has not been well-established. Only a few reports have described residual tumor resection after EGFR-TKI treatment. This report presents two cases of residual tumor resection after a response to EGFR-TKIs for advanced NSCLC harboring EGFR gene mutations. We also review the literature, and discuss the significance of residual tumor resection.

## Case report

### Case 1

**Fig. 1:**
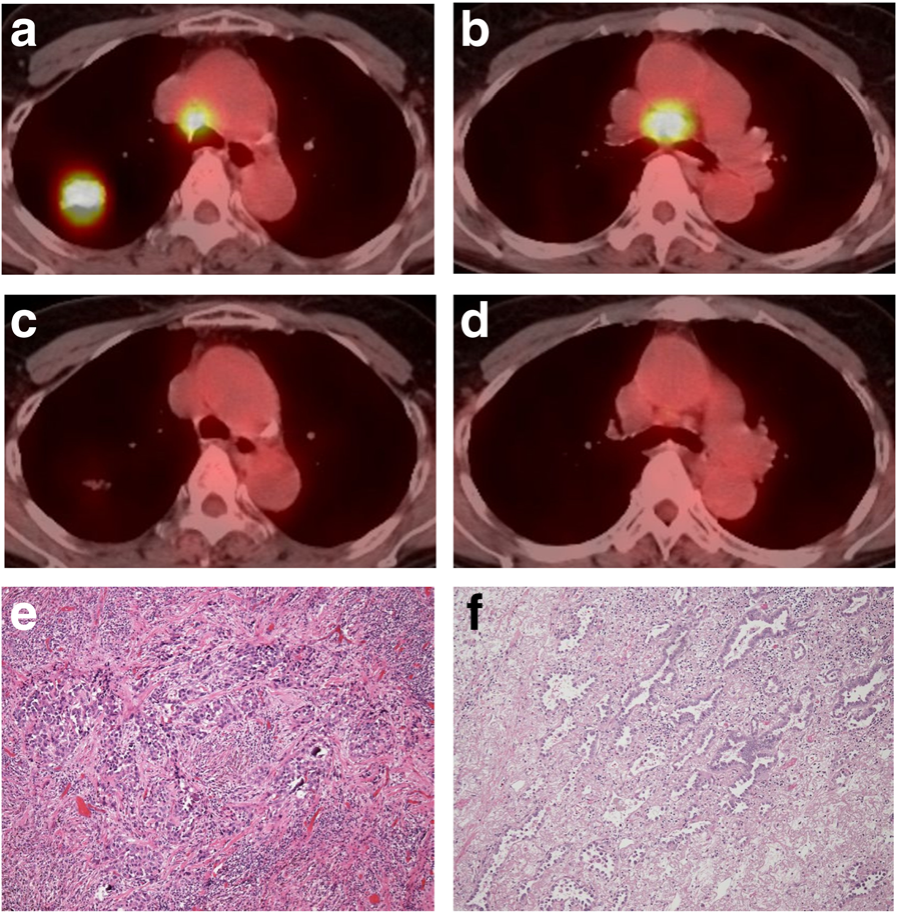
Case 1. PET-CT showing the high fluorodeoyglucose (FDG) uptake of the primary lesion and swelling of the #2 (**a**) and #4 lymph nodes (**b**) before gefitinib therapy. PET-CT showing the regression of the primary lesion and the #2 (**c**) and #4 (**d**) lymph nodes after gefitinib therapy. Histological findings (hematoxylin-eosin staining) showing residual viable tumor cells in the primary lesion (**e**-**f**)

A 72-year-old female asymptomatic non-smoker was referred to our hospital due to an abnormal shadow on chest computed tomography (CT). A physical examination revealed no abnormalities. Her serum CEA level was elevated to 68.9 ng/mL (normal range: <5.0). Chest CT showed a 32-mm solid mass in the right Segment 2 and enlarged right mediastinal lymph nodes (LN station #2, #4). Positron emission tomography/CT (PET/CT) revealed the uptake of fluorodeoxyglucose (FDG) by the pulmonary mass with a maximum standardized uptake value (SUVmax) of 14.1 (Fig. 1a). The uptake of FDG was also observed in the right #2 and #4 LNs (SUVmax 2.6 and 9.6, respectively). She was initially diagnosed with T2aN2M0, stage IIIA adenocarcinoma harboring an EGFR exon 21 L858R mutation via VATS biopsy. She refused to receive any anti-cancer therapy other than EGFR-TKIs. After receiving her informed consent, gefitinib was started as the first-line therapy. Gefitinib was initiated at a dose of 250 mg/day. Following the development grade 3 hepatotoxicity at 6 weeks after the initiation of gefitinib, it was administered 4 days per week. As a result, both the primary tumor and mediastinal lymph nodes showed marked regression. PET/CT also showed a marked metabolic response (Fig. 1c-d). The response was classified as a partial response (PR), according to the Response Evaluation Criteria in Solid Tumors. Her CEA level decreased to 6.3 ng/mL. Although the PR was maintained for 8 months, a further therapeutic effect could not be expected.

We planned to perform tumor resection for the residual intrathoracic lesions. After receiving her informed consent, right upper lobectomy with systemic mediastinal lymph node dissection was performed, 7 days after the discontinuation of gefitinib. Her postoperative course was uneventful. A histological examination showed residual viable tumor cells forming irregularly shaped glands and solid small nests not only in the primary tumor but also in one of the dissected mediastinal lymph nodes (LN station #4) (Ef. 1b, Fig. 1e-f). The residual tumor formed moderately differentiated invasive acinar structures with a lepidic growth area. No vessel permeation was detected on hematoxylin-eosin stained sections. Clinically the tumors were regressed but nuclear atypia in the residual tumor was worse than that in the biopsy specimen obtained before the initiation of gefitinib therapy. An exon 20 point mutation (T790 M), which is known to be associated with EGFR-TKI resistance, was not found in any of these specimens. Gefitinib therapy was continued after discharge. However, she developed multiple brain metastases 8 months after the operation, and stereotactic radiosurgery (SRS) was performed. At 14 months after the operation, she developed extramedullary lumbar spine metastasis. Although afatinib was prescribed and SRS was performed, she died of the disease at 18 months after surgery (29 months after the initial diagnosis).

### Case 2

**Fig. 2:**
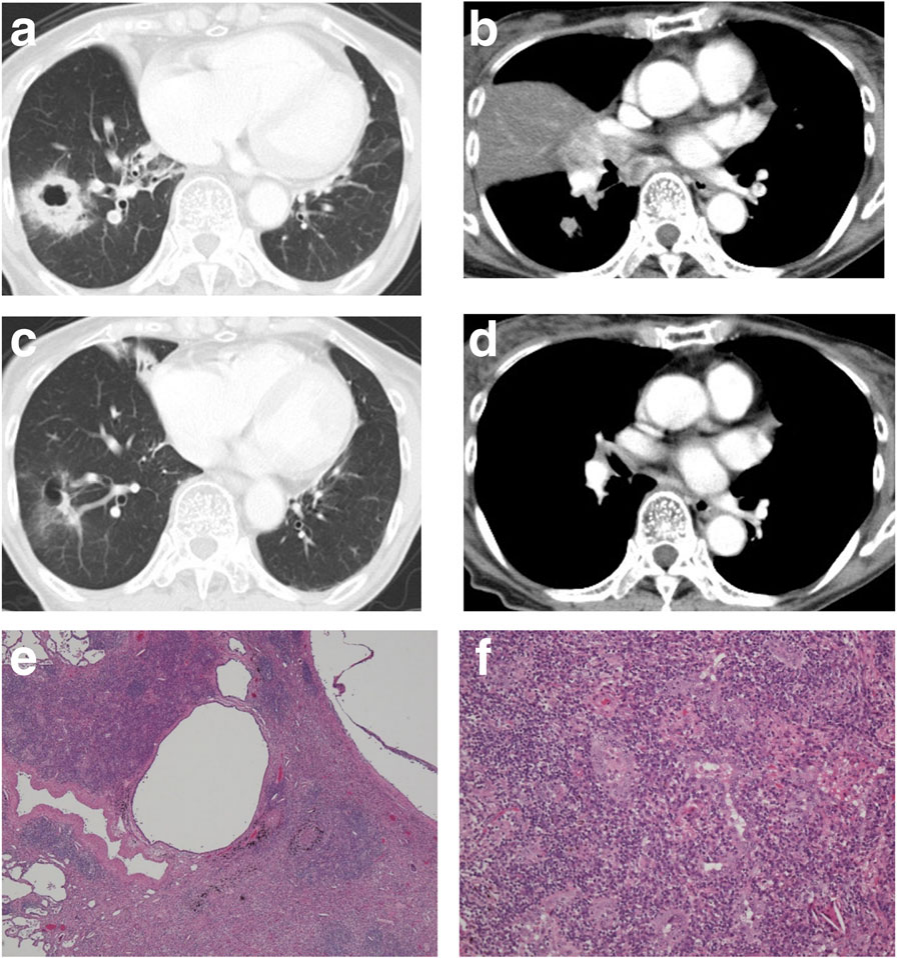
Case 2. Chest CT showing a primary lesion in the right lower lobe (**a**), a 10-mm nodule in the right S6, and right middle lobe atelectasis due to bulky hilar and subcarinal lymph nodes (**b**) before afatinib treatment. Chest CT showing the marked regression of both the primary tumor (**c**) and lymph node metastases, with the disappearance of middle-lobe atelectasis (**d**) after afatinib therapy. Histological findings (hematoxylin-eosin staining) showing the prominent proliferation of fibroblasts (**e**); residual viable tumor cells were found in a small area of the primary lesion (**f**)

A 68-year-old female non-smoker was referred to our emergency department due to progressive dyspnea. On physical examination, she exhibited tachypnea (20 breaths per minute) and her oxygen saturation was 85%, her pulse was regular at 91 beats per minute, and her blood pressure was 116/70 mmHg. Chest CT revealed a 36-mm mass with a cavity in the right S9 (Fig. 2a), and a 10-mm nodule in the right S6. CT also revealed right middle lobe atelectasis due to bulky hilar and subcarinal lymph nodes (Fig. 2b). Concomitantly, a bilateral pulmonary thromboembolism was identified. Transthoracic echocardiography revealed normal a left ventricular function; the right ventricle was dilated with moderate tricuspid regurgitation and her systolic pulmonary artery pressure was elevated (estimated pressure: 55 mmHg); these findings were considered to have been caused by bilateral pulmonary thromboembolism. Ulthrasonography of the lower extremity veins revealed right fibular vein thrombus. Her serum D-dimer, BNP and CEA levels were elevated to 8.0 μg/mL (normal range: <1.0), 332.5 pg/mL (normal range: <18.4) and 14.6 ng/mL (normal range: <5.0), respectively.

Enhanced CT performed 1 week after the administration of intravenous heparin and oral edoxaban tosilate hydrate, which were administered immediately after the detection of the bilateral pulmonary and right fibular vein embolisms, revealed that the pulmonary artery and right fibular venin thrombus had disappeared. Subsequently, the pathological analysis of a bronchoscopy specimen led to a diagnosis of adenocarcinoma harboring an EGFR exon 19 deletion. Based on these results, she was clinically diagnosed with T3N2M0, stage IIIA adenocarcinoma. As lung cancer associated with pulmonary thromboembolism was taken into consideration, EGFR-TKI treatment was proposed as the first-line systemic therapy and afatinib (40 mg/day) was prescribed after receiving her informed consent. In spite of requiring a dose reduction to 30 mg due to grade 3 diarrhea, she became asymptomatic at 2 months after the initiation of treatment. Chest CT revealed the marked response of both tumors and lymph node metastases with the disappearance of the middle lobe atelectasis (Fig. 2c-d). Her serum CEA level decreased to within the normal range (1.4 ng/mL).

We planned surgery to remove the residual lesions. Three days after the discontinuation of afatinib and after receiving her informed consent, we performed right middle and lower lobectomy with systemic mediastinal lymph node dissection. Her postoperative course was uneventful. Complete resection was achieved, with a negative bronchial margin. A histological examination showed the prominent proliferation of fibroblasts accompanied by lymphocytes and histiocytes (Fig. 2e). However, residual viable tumor cells were found in a small area of the primary lesion (Ef.2a, Fig. 2f) because an immunohistochemical examination revealed a few small clusters of atypical cells that were positive anti-pancytokeratin (AE1/3) and thyroid transcription factor 1. There were no malignant cells in the dissected lymph nodes. Down-staging from clinical stage IIIA to pathological stage IA was established. We did not identify an exon 20 point mutation (T790 M) in any of these specimens.

Although afatinib therapy was continued after the operation, she developed spinal cord compression due to 5th cervical vertebral cortical bone metastasis with the onset of neck pain at 8 months after the operation. Direct surgical decompression with tumor debulking and spinal stabilization was performed, followed by radiotherapy. An exon 20 point mutation (T790 M) was found in the resected specimens. Thus, osimertinib is now being prescribed. She is currently alive at 15 months after surgery (18 months after the initial diagnosis).

## Discussion

EGFR-TKIs are effective in more than 70% of cases of advanced NSCLC in patients with TKI-sensitizing EGFR mutations, and are considered to be a first-line treatment of choice for TKI-sensitizing EGFR mutation-positive advanced NSCLC. Treatment naïve patients with such mutations who were treated with EGFR-TKIs have shown longer progression-free survival in comparison to patients treated with chemotherapy; however, they do not show significantly prolonged survival [3].

Whether the resection of a residual tumor after TKI treatment will lead to the prolongation of overall survival in patients with advanced NSCLC patients is unknown. There are a few reports (Table 1) on salvage surgery after a response to EGFR-TKIs in patients with advanced NSCLC; however, the treatment has not been validated from either a surgical or an oncologic point of view. According to our literature survey, 22 cases have been described, most of these are documented in case reports [4–15]. The mean age of the patients was 73.8 years (range: 33 to 78 years). The patients included 2 men and 20 women. The clinical stages at the time of the diagnosis were as follows: Stage IB/IIIA/IIIB/IV 1/6/5/10. Before the operation, 20 patients received gefitinib and 2 received erlotinib. The median duration of EGFR-TKI treatment was 3 months (range, 1 to 36 months). The surgical procedures included extrapleural pneumonectomy (*n* = 1), pneumonectomy (*n* = 3), lobectomy (*n* = 14), and bilobectomy (*n* = 4). Postoperative complications occurred in 2 patients (empyema and chylothorax); no patients died during the perioperative period. Follow-up information was available on 20 patients (range: 1.5 to 94 months). Eleven patients received postoperative therapy (EGFR-TKIs, *n* = 10; chemotherapy *n* = 1). Pathologically, a complete response was achieved in two cases; however, one of the patients developed brain metastasis in spite of continuing gefitinib therapy. The tumor recurred in 11 patients; the median disease-free survival period was 6 months (range: 2 to 28 months). Metastatic tumors were identified in the brain, bone, thorax, and adrenal glands. Five patients died of disease progression at a median of 19 months after surgery (range: 8 to 68 months).

**Table 1:** Report of pulmonary resection of lung cancers in patients after EGFR-TKI treatment

	Age	Sex	TNM classification	Initial Stage	TKI	Surgical procedure	Pathological outcome	Complication	Outcome
Takamochi et al. 2007 [5]	73	F	cT2N2M1	IV	gefitinib	bilobectomy	pT1N0M1		no recurrence (2 months)
	57	F	cT1N2M0	IIIA	gefitinib	lobectomy	CR	chylothorax	no recurrence (1.5 months)
Kappers et al. 2008 [6]	67	F		IIIA	erlotinib	lobectomy	near CR		unknown
Levchenko EV et al. 2009 [7]	62	F		IV	erlotinib	bilobectomy	EF 1a		meta in thorax (15 months)
	48	M		IV	gefitinib	lobectomy	residual viable tumor		adrenal meta (5 months), DWD (8 months)
Shen et al. 2010 [9]	73	F	cT2N3M0	IIIB	gefitinib	lobectomy	alveolar cell carcinoma, N0		no recurrence (20 months)
Hishida et al. 2010 [8]	73	F	cT4N2M0	IIIB	gefitinib	lobectomy	pT2N1M0		bone meta (6 months), DWD (17 months)
	63	F	cT2N3M1	IV	gefitinib	lobectomy	pT1N2M0		brain meta (5 months), AWD (24 months)
	33	F	cT4N0M0	IIIA	gefitinib	extra pleural pneumonectomy	pT4N2M0		brain meta (3 months), DWD (19 months)
	54	M	cT4N3M1	IV	gefitinib	lobectomy	pT4N0M0		meta in thorax (6 months), AWD (10 months)
	71	F	cT2N3M0	IIIB	gefitinib	lobectomy	pT2N2M0		meta in thorax (4 months), DWD (21 months)
	57	F	cT4N0M1	IV	gefitinib	lobectomy	pT2N0M0		unknown
Liu et al. 2011 [10]	64	F	cT2bN2M1	IV	gefitinib	bilobectomy	CR		no recurrence (21 months) Hashimoto et
Hashimoto et al. 2012 [12]	66	F	cT4N1M1a	IV	gefitinib	lobectomy	pT2aN0M0 StageIB		no recurrence (12 months)
Ong et al. 2012 [11]	78	F	cT3N2M0	IIIA	erlotinib	lobectomy	residual viable tumor, N0		meta in thorax (10 months)
Funakoshi et al. 2012 [14]	69	F	cT2aN0M0	IB	gefitinib	pneumonectomy	pT2aN0M0 StageIB	empysma	no recurrence (26 months)
Marech et al. 2013 [13]	67	F	cT4N2M0	IIIB	gefitinib	lobectomy	pT1N0M0		no recurrence (7 months)
Lopez-Gonzales et al. 2013 [15]	66	F	cT4N2M0	IIIB	gefitinib	pneumonectomy	<10% of residual viable tumor		brain meta (17 months), AWD (22 months)
Hishida et al. 2014 [4]	62	F	cT2aN2M0	IIIA	gefitinib	lobectomy	EF 1a		no recurrence (36 months)
	51	F	cT2N3M1	IV	gefitinib	lobectomy	pT1N0M0, EF0		brain meta (2 months), AWD (94 months)
	58	F	cT1bN0M1a	IV	gefitinib	bilobectomy	pT1N1M0, EF1a		meta in thorax (14.4 months), AWD (63 months)
	58	F	cT2N2M0	IIIA	gefitinib	pneumonectomy	CR		brain meta (28 months), DWD (68 months)
Our two cases. 2017	72	F	cT2N2M0	IIIA	gefitinib	lobectomy	pT1N2M0, EF 1b		brain meta (8 months), DWD (18 months)
	68	F	cT3N2M0	IIIA	afatinib	bilobectomy	pT1N0M0, EF 2a		bone meta (8 months), AWD (14 months)

*DWD* died with disease, *AWD* alive with disease, *CR* complete response

In our two cases, surgery was performed without any complications. However, there were some serious problems regarding the oncological outcomes. Because our patients had bulky N2 disease, initial surgery would not offer a survival advantage and complete resection would not be technically feasible. Their tumors were TKI-sensitizing EGFR mutation-positive and showed a marked regression after a response to EGFR-TKI; complete resection was subsequently achieved. In the two present cases, the tumor cells were not eradicated in spite of the marked response to EGFR-TKIs and both our patients relapsed. In Case 1, gefitinib could not downstage the disease and failed to facilitate long-term disease-free survival despite the continuous administration of postoperative gefitinib therapy. She developed brain and leptomeningeal metastases after the surgery. We successfully treated Case 2 by the simultaneous administration of anticoagulants and afatinib, with marked tumor regression and the complete remission of bilateral pulmonary and right fibular vein thrombus which were considered to have been caused by lung cancer. Cancer activates the coagulation systems through multiple mechanisms leading to the development of a prothrombotic state. Patients with a central venous catheter, those receiving chemotherapy and those undergoing surgical treatment are at increased risk of vascular thrombosis. Thrombus has not been reported as an adverse event in patients receiving EGFR-TKIs. We therefore proposed EGFR-TKIs as a first-line systemic treatment. According to the results of a recent phase II, randomized controlled trial (LUX-Lung 7), afatinib significantly improved progression-free survival and time-to-treatment failure in treatment-naïve patients with such mutations in comparison to gefitinib [16]. We therefore, chose afatinib as a timely drug for first-line treatment in Case 2. Afatinib was able to downstage the disease in the patient, but failed to facilitate long-term disease-free survival despite the continuous administration of afatinib after surgery. She developed cervical vertebral cortical bone metastasis with compression of the spinal cord after surgery.

EGFR-TKI monotherapy may be unable to completely cure advanced NSCLC. Hishida et al. reported that the mechanism of EGFR-TKIs is cytostatic rather than cytotoxic, and EGFR-TKIs could not eradicate micrometastatic tumor cells even after a marked clinical response [4]. We did not find the acquired resistance gene (the exon 20 point mutation [T790 M]), in the initially resected specimens in our two cases. However, in Case 2, a tumor specimen obtained from vertebral cortical bone metastasis expressed T790 M. To the best of our knowledge, this is the first reported case of surgery for advanced NSCLC after a response to afatinib.

The optimal timing and validity of salvage surgery for residual lesions when TKIs are offered to advanced NSCLC patients with the driver gene mutation remain unclear. Recent studies have reported that the addition of local consolidative therapy including radiation and surgery after initial systemic therapy was feasible and led to good local control and a significantly extended progression-free survival time in comparison to maintenance treatment [17, 18]. Our limited data also suggest that good local control at the primary site might be established by residual tumor resection. Promising therapeutic strategies are being developed to overcome various forms of acquired resistance due to heterogeneous mechanisms. The biological information obtained from repeated biopsy or recently available liquid samples (i.e. blood) should be used to understand these heterogeneous mechanisms. More clinical data will be needed to further investigate the role of surgery during molecular-targeted therapy for advanced NSCLC.

## Conclusion

The timing and validity of salvage surgery for residual lesions remain unclear when TKIs are prescribed to advanced NSCLC patients with driver gene mutations. In our two cases, surgery was performed without any complications and preoperative EGFR-TKI therapy made it possible to achieve complete resection. Surgical resection of the residual tumor might contribute to good local control but there are some serious problems regarding the oncological outcomes. More clinical data will be needed to further investigate the role of surgery in patients with advanced NSCLC harboring EGFR gene mutations.

## Abbreviations

AWD: Alive with disease
CEA: Carcinoembryonic antigen
CR: Complete response
DWD: Died with disease
EGFR: Epidermal growth factor receptor
NSCLC: Non-small cell lung cancer
PR: Partial response
TKI: Tyrosine kinase inhibitor
